# Arterialized oxygen tension and unfavorable clinical outcomes in pediatric cystic fibrosis

**DOI:** 10.3389/fped.2025.1653323

**Published:** 2025-10-08

**Authors:** René Gaupmann, Saskia Gruber, Klara Schmidthaler, Bianca Pauger, Brigitte Mersi, Alexandra Graf, Zsolt Szépfalusi, Sabine Renner, Eleonora Dehlink

**Affiliations:** ^1^ Department of Pediatrics and Adolescent Medicine, Division of Pediatric Pulmonology, Allergology and Endocrinology, Medical University of Vienna, Vienna, Austria; ^2^ Institute for Medical Statistics, Centre for Medical Data Science, Medical University of Vienna, Vienna, Austria

**Keywords:** blood gas monitoring, respiratory function test, disease progression, secondary prevention, risk stratification

## Abstract

**Introduction:**

Maintaining good lung function is a primary goal in managing Cystic Fibrosis (CF). As spirometry lacks sensitivity for detecting mild lung disease, early progression often remains unrecognized. To overcome this limitation, more sensitive monitoring tools are needed. We evaluated arterialized oxygen tension (pO_2_) as an easily accessible, and widely applicable surveillance method.

**Methods:**

In this retrospective observational single-center cohort study, arterialized gas exchange was assessed in 103 young people with CF (47 females, 56 males, aged 5–18 years). Trends from baseline (age 5 years) to early adulthood and performance relative to annual best pulmonary function (FEV1) and lung clearance index were examined, along with baseline oxygen tension's predictive value on future FEV1 decline and the occurrence of CF-related complications.

**Results:**

pO_2_ correlated significantly with FEV1 (*p* < 0.001) and inversely with lung clearance index (*p* < 0.001). Higher pO₂ was associated with pancreatic sufficiency (*p* = 0.069) and dual CFTR modulator use (*p* < 0.05), with no differences by sex or chronic Pseudomonas aeruginosa infection. By age 5, 19.8% of young individuals with CF had pO₂ below 80 mmHg (5th percentile), of whom 73.7% had normal FEV1. A linear mixed model showed a steeper FEV1 decline in those with abnormal pO_2_ at baseline [estimate = 0.06 (Z-score*year^−1^), *p* < 0.001]. Early low pO_2_ was significantly associated with a higher probability of allergic bronchopulmonary aspergillosis (HR = 7.69, *p* = 0.016) and a trend towards early CF-related diabetes (HR = 2.78, *p* = 0.06) and early chronic Pseudomonas aeruginosa infection (HR = 2.38, *p* = 0.09).

**Conclusions:**

Early abnormal pO_2_ at age 5 significantly correlated with accelerated FEV1 decline and a greater probability for CF-related complications. Implementing arterialized oxygen tension may offer valuable insights beyond spirometry alone in identifying high-risk patients.

## 1 Introduction

Cystic Fibrosis (CF) is an autosomal recessive disorder affecting approximately 1 in 3,500 Caucasian newborns (1). It results from a dysfunction in the CF transmembrane conductance regulator (CFTR) protein, causing thickened mucus and impaired airway clearance (2). Structural lung changes begin early, even in infancy, well before clinical symptoms appear or spirometry can detect them (3, 4). Early diagnosis and intervention are crucial to interrupt the cycle of inflammation, infection, and lung damage, thereby mitigating disease progression (3–5). Early identification of high-risk individuals allows early intervention to optimize long-term outcomes (5).

Spirometry is the preferred method for monitoring CF-related lung disease progression (6, 7). However, spirometry has certain limitations. Firstly, it can only be performed from a certain age, typically school age and older, as it relies on adequate cooperation (8). Secondly, spirometry lacks sensitivity in detecting very early or mild lung disease (3). Alternative techniques, such as computed tomography or magnetic resonance imaging of the chest or multiple breath washout, may identify structural or functional lung changes earlier in the disease course than spirometry. However, these alternatives have associated concerns, including radiation exposure, the need for anesthesia to prevent motion artifacts, and limitations in availability and costs (9). Thus, the optimal methods for detecting early lung parenchymal and functional changes remain unclear (3).

As the lungs' primary role is oxygen uptake, measuring arterial oxygen tension appears to be a viable option for further exploration (10). While pulse oximetry is non-invasive, its correlation with arterial oxygen tension is poor (11). In pediatric settings, arterial puncture is impractical, but arterialized capillary blood sampling is a reliable alternative. Arterialized capillary oxygen tension (pO_2_) offers a highly accurate estimation, as a suitable substitute for true arterial oxygen levels (11–13). Kraemer et al. investigated longitudinal pO_2_ progression in young people with CF (PwCF) and found that pO_2_ trends paralleled those of FEV1, with both showing a decline until early adulthood (14, 15). Since low FEV1 at a young age is associated with a more severe clinical course in PwCF, we hypothesized that pO_2_ might follow a similar pattern (5).

This retrospective observational study evaluated the role of pO_2_ as an additional marker for pediatric CF lung disease and its potential for risk stratification, focusing on whether low pO_2_ at a young age is associated with a worse clinical course.

## 2 Methods

### 2.1 Study design and population

This retrospective observational single-center cohort study included individuals newly diagnosed with CF after the nationwide newborn screening program's implementation in October 1997, until the analysis in December 2020. Eligible PwCF were under the care of the pediatric CF center at the Medical University of Vienna.

PwCF had to be over 5 years of age at enrolment, with their annual best pulmonary function tests (best FEV1), concurrently sampled arterialized capillary blood gases and multiple breath washout maneuvers (MBW) reviewed up to the age of 18. PwCF under 5 years of age who might be unable to perform spirometry accurately, and those after solid organ transplantation were excluded from analysis. Associations were analyzed for pO_2_, FEV1, and LCI_2.5_ when available (introduced in 2015). To evaluate the potential for risk stratification and prediction of future clinical outcomes, we analyzed the longitudinal progression of FEV1 and the time to onset of CF-related complications—previously shown to be associated with an unfavorable disease course—stratified by dichotomized pO₂ at age 5 (normal vs. abnormal). All necessary data were extracted from medical records. An explanatory flowchart is shown in Supplementary Figure 1 in the online supplement.

The study protocol was approved by the local ethics committee, adhering to the Declaration of Helsinki and Good Clinical Practice. Due to the retrospective study design, no informed consent was required before the medical records were reviewed.

### 2.2 Pulmonary function tests

Spirometry followed the European Respiratory Society guidelines using the MasterScreen Body (Vyaire Medical, Mettawa, Illinois, USA, Software: Sentry Suite) (16). Forced expiratory volume in the first second (FEV1) was reported in Z-scores using the Global Lung Initiative normative dataset (17).

The lung clearance index (LCI_2.5_) was derived from nitrogen multiple breath washout using the ExhalyzerD system (EcoMedics, Duernten, Switzerland, Software: Spiroware Version 3.2.2), adhering to the standardized protocol outlined in the consensus statement (18). LCI_2.5_ has been routinely obtained at our CF center since 2015. As introduced by Anagnostopoulou et al., 7.91 was considered the upper limit of normal (19, 20).

### 2.3 Capillary blood sampling and blood gas analysis

Blood gas sampling from the earlobe preceded pulmonary function tests at every outpatient visit starting from the third spirometry, typically around the age of 5 years. Local capillary blood circulation was increased using topical nonyl vanillamide and nicotinic acid-β-butoxyethyl ester containing ointment (Finalgon®, Sanofi, Paris, France). After 15 min incubation time, capillary blood was collected in heparinized plastic capillaries and immediately analyzed for oxygen tension (pO_2_) in mmHg at room temperature using an ABL800 FLEX blood gas analyzer (Radiometer Medical ApS, Copenhagen, Denmark). The analyzer was located in the same room, ensuring a short transfer time. pO_2_ values were converted into *Z*-scores based on pediatric normative data from Gaultier et al. (13). The lower limit of normal (LLN) for pO_2_ was defined as the 5th percentile of a healthy pediatric population, corresponding to a *Z*-score of −1.64 (5–8 years: 80 mmHg; ≥8 years: 84.3 mmHg).

The sampling technique was highly accepted, with a 100% acceptance rate among all 5-year-olds included in the study.

### 2.4 Definition of CF-related complications

The modified Leeds criteria were applied to define chronic Pseudomonas aeruginosa (PsA) airway infection using surveillance microbiology airway samples (21). CF-related diabetes (CFRD) was defined by a pathological oral glucose tolerance test (OGTT), routinely performed annually from the age of 10 during routine follow-ups when patients were in a stable health condition (6). Allergic bronchopulmonary Aspergillosis (ABPA) episodes were diagnosed based on the Cystic Fibrosis Foundation Consensus, as described elsewhere (22).

### 2.5 Statistics

Data analysis was conducted using R software, version 4.3.2 (The R Foundation for Statistical Computing, Vienna, Austria). Descriptive statistics included absolute numbers and percentages for categorical data, while quantitative parameters (ages 5–18) were presented as mean ± SD. Linear mixed models with a random patient effect were used to assess differences and the progression of serial measured variables. To evaluate CFTR modulator effects, a binary factor was included to compare measurements before vs. after modulator initiation.

To assess the impact of pO_2_ at age 5 on the future clinical course, the dataset was split into two groups: pO_2_ *Z*-score ≤1.64 vs. pO_2_ *Z*-score ≥−1.64. Baseline data were compared using *t*-tests for continuous variables and Fisher's exact tests for categorical data. The future FEV1 time course was analyzed using a two-step approach: (1) simple linear mixed-effect models with random patient effects for our baseline grouping variable and potential confounders [age, year of birth, sex, pancreatic insufficiency, genotype, baseline FEV1, body mass index (BMI) at age 5, first and chronic PsA infection before age 5, chronic Staphylococcus aureus infection before age 5 and CFTR modulator therapy], followed by (2) a multivariable linear mixed-effect model, including all significant factors from the simple models (more detailed in the online supplements' statistics section) (5). To investigate the impact of early hypoxemic states on the probability for CF-related diseases (ABPA, CFRD, chronic PsA infection), Kaplan–Meier and univariable Cox regression models were employed. The level of significance was set to <0.05.

## 3 Results

### 3.1 Study population

Among 150 eligible young PwCF, 47 were excluded. Of the remaining 103 (47 females and 56 males) aged 5–18, 923 arterialized blood gases were reviewed. Patient numbers and examinations by age group are detailed in Supplementary Table 1 in the online supplement. The mean observational period was 8.1 ± 4.0 years. Of the 103 patients, 87 (84.5%) were pancreatic-insufficient, 29 (28.1%) received CFTR modulator therapy at some point during the observational period; none received modulators before the age of 6 years. 10/103 (9.7%) patients were considered chronically infected with PsA and one patient was diagnosed with ABPA as early as age 5. A detailed overview of patients' characteristics at baseline is provided in Table 1 (23).

**Table 1:** Patients’ characteristics.

Patients under observation (N)	103
Blood gas samples in total (N)	923
Blood gas samples per patient^a^	9 (1–14)
SaO_2_ ≥ 93% at sampling timepoint (N)	923 (100%)
Observational period [years]^b^	8.1 (4.0)
Sex	
Female	47 (45.6%)
Male	56 (54.4%)
Genotype	
high-risk	86 (83.5%)
low-risk	10 (9.7%)
Unknown	7 (6.8%)
Pancreas insufficiency	87 (84.5%)
CFTR modulator therapy at any time during the observation	29 (28.1%)
Dual CFTR modulator therapy^c^	26/29 (89.7%)
HEMT^d^	3/29 (10.3%)
chronic PsA infection ≤5 years	10 (9.7%)
ABPA ≤ 5 years	1 (0.97%)
CFRD ≤ 5 years	0 (0%)

If not otherwise indicated the number of patients & proportion [n (%)] were reported.

^a^ Median (range).

^b^ Mean (standard deviation).

^c^ Ivacaftor/lumacaftor or ivacaftor/tezacaftor.

^d^ Ivacaftor monotherapy or elexacaftor/tezacaftor/ivacaftor.

Risk stratification within genotypes was determined based on CFTR mutations and functional phenotypes. CFTR mutations were categorized as high-risk if both alleles featured class I, class II, or class III mutations, while class IV and V mutations were classified as low-risk; otherwise, they were designated as unknown (23).

ABPA, allergic bronchopulmonary aspergillosis; CFRD, CF-related diabetes; CFTR, cystic fibrosis transmembrane conductance regulator; HEMT, highly effective modulator therapy; N, number of patients; PsA, pseudomonas aeruginosa; SaO_2_, arterialized oxygen saturation.

### 3.2 Pulmonary function and gas exchange during childhood and adolescence

**Figure 1:**
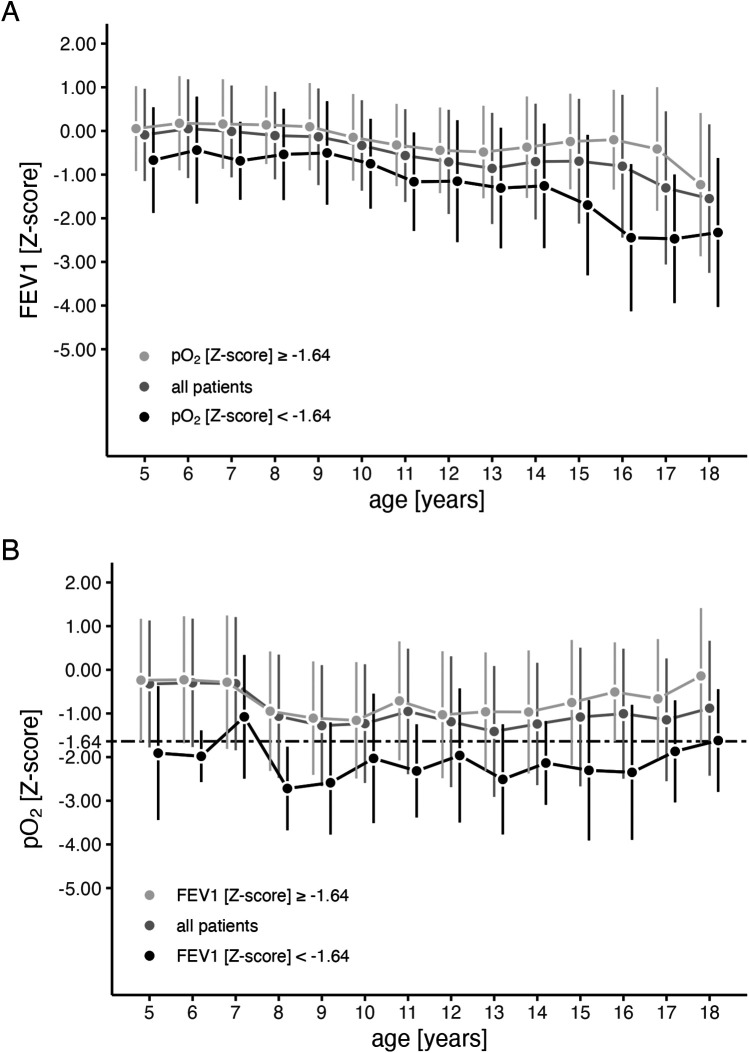
Progression of arterialized oxygen tension (pO_2_) and FEV1 during childhood and adolescence. Mean values (points) and standard deviations (vertical lines) for FEV1 **(A)**, and pO_2_ **(B)** are shown across age groups for all patients (grey) and stratified by whether the corresponding pO_2_ **(A)** or FEV1 **(B)** falls above (light grey) or below (black) −1.64 *Z*-scores. Mean values were calculated based on the actual number of patients in each age group. The number of patients is detailed in Supplementary Table 1 in the online supplement.

Within the study cohort, pulmonary function (FEV1) showed a significant decline from early childhood to adulthood (*p* < 0.001, Figure 1A). While the mean arterialized pO_2_ remained stable above the lower limit of normal (*Z*-score of −1.64) across all age groups, lower pO_2_ values were observed in PwCF with lower FEV1 values (*p* < 0.001, Figure 1B). For further analysis, PwCF with pO_2_ below a Z-score of −1.64 were classified as “hypoxemic”, while those with values equal to or above −1.64 were classified as “normoxemic”.

Pancreatic sufficient PwCF tended to have higher average pO₂ values across all age groups (*p* = 0.069), while levels did not differ by sex or chronic PsA airway infection (defined for each age group in Supplementary Table 1, online supplement), as illustrated in Supplementary Figure 2 (online supplement).

### 3.3 pO_2_ in relation to LCI_2.5_ and FEV1

**Figure 2:**
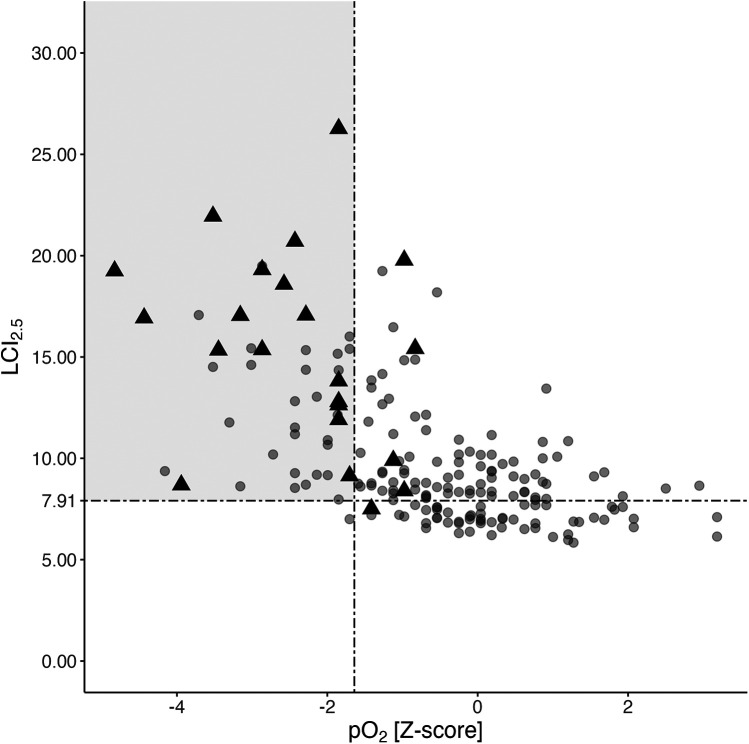
Association of corresponding LCI_2.5_, pO_2_, and FEV1 values. Distribution of LCI_2.5_ and pO_2_ values from a total of 188 N_2_BMW maneuvers and corresponding blood gas analyses from 85 PwCF aged 6 to 18 years (see Supplementary Table 1 in the online supplement for age distribution). Black triangles represent individuals with poor pulmonary function (FEV1 *Z*-scores <−1.64), whereas the grey circles indicate individuals with normal FEV1 at the time of assessment. The grey rectangle indicates a pathologically high LCI_2.5_ (>7.91) combined with a concurrently low pO_2_ *Z*-score (<−1.64). FEV1, forced expiratory volume in the first second; LCI_2.5_, lung clearance index; N_2_MBW, nitrogen multiple breath washout; pO_2_, arterialized oxygen tension; PwCF, people with CF.

A total of 188 nitrogen multiple breath washout maneuvers from 85 young PwCF, spanning ages 6–18 years and obtained concurrently with blood gas sampling and spirometry, were analyzed (more detailed in Supplementary Table 1 in the online supplement). Lower pO_2_ values were associated with higher LCI_2.5_ across all ages (Figure 2, linear mixed model: estimate −1.09, 95%−CI: −1.38 to −0.81, *p* < 0.001). LCI_2.5_ was pathologically high in 133 out of 188 measurements, with 45 (33.8%) showing hypoxemia in arterialized blood gases, while FEV1 *Z*-score being below −1.64 in only 21 (15.8%) cases (Figure 2). Forty-four out of 45 PwCF (98%) with hypoxemia had an LCI_2.5_ exceeding 7.91, while only one individual hat a normal LCI_2.5_ (2%).

### 3.4 pO_2_ at the age of 5 years

Seven out of the total cohort of 103 PwCF lacked sufficient data before the age of 5 years, either due to late diagnosis or because they transferred to our center at later ages. Among the remaining 96 patients at 5 years of age, the mean FEV1 *Z*-score was −0.09 ± 1.056 (SD), corresponding to 98.8 ± 13.5% predicted. Supplementary Table 2 in the online data supplement provides a more detailed overview of patients' characteristics at the age of 5 years.

**Figure 3:**
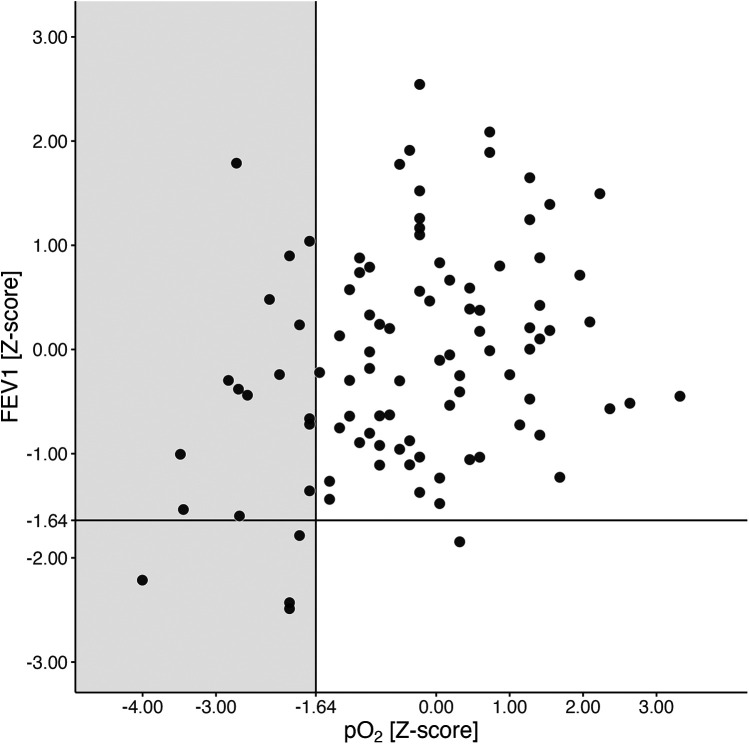
FEV1 and pO_2_ at the age of 5 years. pO_2_ and FEV1 *Z*-scores at the age of 5 years are shown. The black lines represent the lower limits of normal (LLN) for both pO_2_ and FEV1 *Z*-scores. The grey rectangle indicates hypoxemic 5-year-olds (pO_2_ *Z*-score <−1.64, equivalent to 80 mmHg). Abbreviations used: FEV1 (forced expiratory volume in the first second), N (number of patients), pO_2_ (arterialized oxygen tension), PwCF (people with CF).

Five of 96 (5.2%) had FEV1 values below the lower limit of normal, while 19 of 96 (19.8%) were already hypoxemic at this early age (as depicted in Figure 3). Compared to normoxemic PwCF, the hypoxemic group tended to be female, with 12 out of 19 (63.2%, *p* = 0.091), and had significantly lower FEV1 values (*p* = 0.021, see Supplementary Table 2 in the online data supplement).

### 3.5 Impact of CFTR modulator therapy on FEV1 and pO_2_ trend

**Figure 4:**
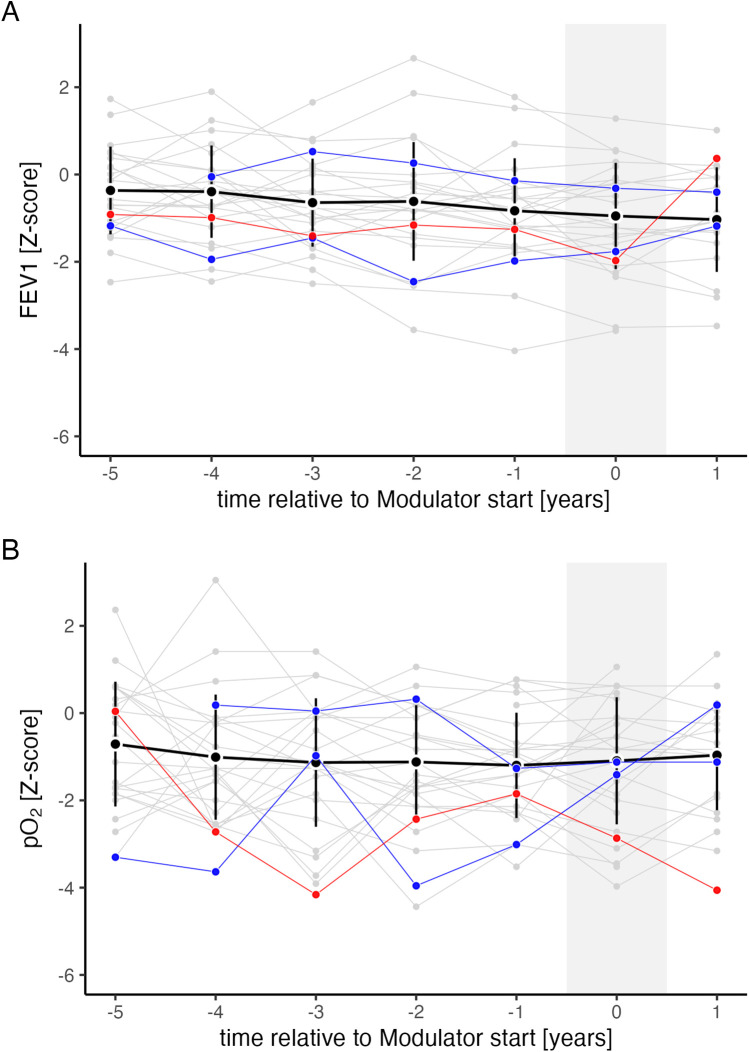
FEV1 and pO_2_ trend relative to CFTR modulator introduction. Progression of FEV1 **(A)** and pO₂ **(B)** relative to CFTR modulator introduction, expressed in years before (−) and after () initiation. Shown are mean values (black points) and standard deviations (black vertical lines) for all individuals on ivacaftor/lumacaftor or ivacaftor/tezacaftor. Individual trajectories are shown separately for each person with CF (PwCF) on ivacaftor/lumacaftor or ivacaftor/tezacaftor (grey, *n* = 26), ivacaftor monotherapy (blue, *n* = 2), and the triple combination of elexacaftor/tezacaftor/ivacaftor (red, *n* = 1).

The proportion of PwCF receiving CFTR modulator therapy increased with age, ranging from 7% to 25% among patients aged 10–18 years (Supplementary Table 1, online supplement). Figure 4 shows FEV1 and pO₂ trends before and after CFTR modulator initiation. Among individuals on dual CFTR modulators (ivacaftor/lumacaftor or ivacaftor/tezacaftor), no significant change in FEV1 was observed (estimate: −0.56; 95% CI: −4.23 to 3.11; n.s.), whereas pO₂ showed a slight improvement (estimate: 0.46; 95% CI: 0.01–0.91; *p* = 0.044). For the three PwCF on Highly Effective Modulator Therapy (HEMT), statistical analysis was not performed due to the small sample size; these patients were followed only until modulator initiation in the prediction model.

### 3.6 Association between early low pO_2_ and clinical course

**Figure 5:**
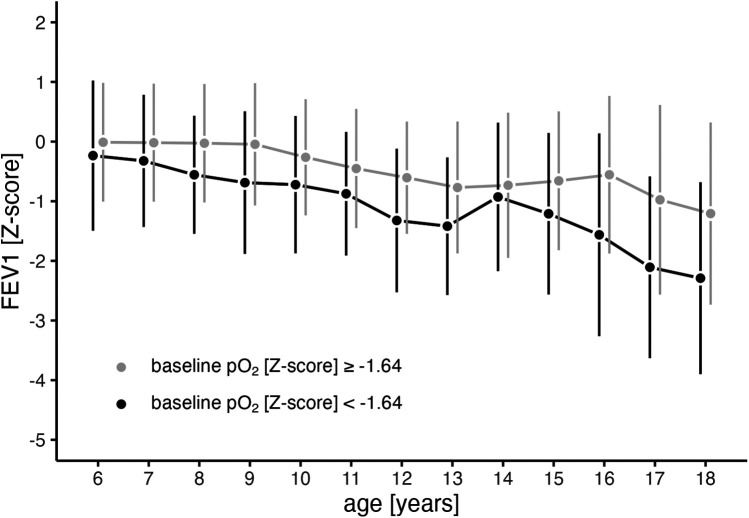
Trajectory of FEV1 in relation to pO_2_ at 5 years (baseline). Mean values (points) and standard deviations (vertical lines) for FEV1 progression from ages 6 to 18 years are shown separately for individuals with abnormal pO_2_ *Z*-score (<−1.64, black line) and those with normal pO_2_ *Z*-score (≥−1.64, grey line) at baseline (age 5). At age 5, a pO_2_ *Z*-score of −1.64 corresponds to 80 mmHg. FEV1 (forced expiratory volume in the first second), pO_2_ (arterialized oxygen tension).

Being hypoxemic at the age of 5 years was significantly associated with future FEV1 loss within our study. Compared to individuals with normal pO_2_, *Z*-scores below −1.64 (equivalent to 80 mmHg) at this early stage were associated with a subsequent steeper decline in FEV1 (*p* < 0.001, estimate = −0.06, 95%−CI: −0.09 to −0.02, Figure 5). This association remained significant even in the multivariable model, adjusted for other significant influencing factors on the FEV1 course from the simple models, such as genotype, pancreatic insufficiency, chronic PsA infection before age 5, FEV1 at baseline, year of birth, and CFTR modulator use (calculations and estimates are provided in the statistics part of the online supplement and Supplementary Table 4).

The number of observed CF-related complications during the observational period is provided in Supplementary Table 3 in the online supplement. Five-year-olds with hypoxemia in arterialized blood gases were significantly more likely to experience an ABPA episode compared to those with normoxemia [HR: 7.69, 95% CI: 2.22–25, *p* = 0.001; see Supplementary Figure 3A in the online supplement]. A similar trend was observed for the onset of CFRD and the acquisition of chronic PsA infections in hypoxemic states at age 5 [CFRD: HR: 2.78, 95%−CI: 0.96–7.69, *p* = 0.06; PsA: HR: 2.38, 95%−CI: 0.87–6.67, *p* = 0.09; see Supplementary Figures 3B, C in the online supplement].

## 4 Discussion

In this study, we assessed pO_2_ in arterialized blood as an easily accessible, and widely applicable method for monitoring lung disease and identifying patients at risk of a more severe disease course. In the analyzed CF cohort, pO_2_ remained stable from 5 to 18 years, which contrasts with the longitudinal decline in FEV1 observed in young PwCF. Dual CFTR modulators positively influenced the pO₂ trajectory, whereas its levels were independent of pancreatic function, sex, and chronic Pseudomonas aeruginosa infection. While most children (94.8%) had normal lung function at age 5, 19.8% already showed abnormally low pO_2_. These children experienced a greater decline in FEV1 during childhood and adolescence and faced a higher risk of CF-related complications such as ABPA, CFRD, and chronic PsA infections.

The longitudinal pO_2_ progression in our study diverges from previously published data on pO_2_ (14, 15). In the study of pediatric PwCF by Kraemer and colleagues, pO_2_ values were notably lower and exhibited a linear decline during childhood, starting at 80.7 ± 1.9 mmHg at 5 years of age and decreasing to a mean of 69.9 ± 1.6 mmHg at age 18 years (14). This discrepancy may be attributed mainly to the inclusion of pre-symptomatic children through newborn screening in the present study, the generally improved overall health of individuals with CF in recent years, and the use of CFTR modulators. The latter may also partially explain the slightly increasing pO₂ trend observed in our older adolescents. To our knowledge, this is the first study to evaluate the impact of any generation of CFTR modulator therapy on pO₂.

In pediatrics, hypoxemia is rarely defined by arterial or arterialized pO_2_; instead, it is more commonly characterized as transcutaneous oxygen saturation falling below 90% or 93%, depending on the child's age (24). Notably, within our cohort, even PwCF having hypoxemic pO_2_ values exhibited arterialized oxygen saturation above 93%. We did not measure transcutaneously, but there is a strong correlation between arterial oxygen saturation and transcutaneous oxygen saturation (25). Abnormal pO₂ despite normal oxygen saturation aligns with the physiology of the oxygen-hemoglobin dissociation curve, which flattens at higher pO₂ levels. Even when pO₂ falls below the lower limit of normal (5th percentile), oxygen saturation may remain within the normal range (26, 27). We therefore propose arterialized pO₂ as a more accurate surrogate marker of oxygenation than oxygen saturation.

Predicting disease progression is crucial for identifying PwCF who may benefit from more aggressive surveillance strategies or therapeutic interventions (5, 28). FEV1 remains the most significant predictor of mortality and early CF lung disease progression (5). However, especially in the era of highly potent CFTR modulators, the proportion of patients with pulmonary function within the normal range is increasing (29). FEV1 alone may not provide a comprehensive assessment of lung damage, particularly in cases of milder CF lung disease or during the subclinical period from birth to school age, when disease progression can occur without noticeable symptoms and spirometry is unreliable (3, 4, 30, 31). While we cannot draw conclusions for the subclinical period of infancy as children under 5 years were excluded from this study, pO₂ may offer significant advantages as an adjunct to traditional spirometry, particularly in mild CF lung disease.

Blood oxygen tension primarily depends on oxygen uptake and the lung's ability to transfer oxygen into the bloodstream independent of factors such as sex, age, anthropometrics and ethnicity (10, 32). Ventilatory inhomogeneity and the simultaneous increase in dead space ventilation may play a significant role in gas exchange abnormalities among pediatric PwCF (14, 31). Over the last decade, multiple breath washout, serving as a marker for ventilatory inhomogeneity, has emerged as a promising method for monitoring early or mild CF lung disease (33). Not only does it serve as a more sensitive marker for early structural lung damage compared to FEV1, but abnormal LCI_2.5_ in early childhood have also been demonstrated to predict poor pulmonary function during school years (3, 34, 35). In our study, low pO_2_ was associated with high LCI_2.5_, suggesting a potential indication of poor oxygenation due to ventilatory inhomogeneity. Moreover, low pO_2_ showed greater sensitivity in detecting LCI_2.5_ values above 7.9 compared to low FEV1, with sensitivity increasing from 0.18 for FEV1 to 0.45 for pO_2_. This suggests that blood gas analysis could be a more sensitive method for detecting mild CF lung disease than spirometry.

Limitations of our study are the limited generalizability of our results due to the retrospective design of the study. Additionally, outcomes may have been influenced by the evolving landscape of new treatment approaches, particularly the widespread availability of Highly Effective Modulator Therapy over the last years, especially among young children. Our findings must be confirmed in prospective studies involving PwCF on triple CFTR modulator therapy. This is particularly relevant as most PwCF on triple therapy have normal FEV1 (29).

While we do not advocate the use of pO_2_ as a direct substitute for LCI_2.5_ or spirometry, it is worth noting that multiple breath washouts can be laborious and resource-intensive, particularly in very young children (35). Our data suggest that pO_2_ is a more sensitive marker than FEV1 for mild CF lung disease and, hence, could serve as an easily accessible, and widely applicable addition to established monitoring tools to identify young PwCF at increased risk of a more severe disease.

## Data Availability

The original contributions presented in the study are included in the article/Supplementary Material, further inquiries can be directed to the corresponding author.

## References

[1] Frischer T Eber E Ellemunter H Zacharasiewicz A Kaluza I Riedler J Cystic fibrosis in Austria. Wien Klin Wochenschr. (2017) 129(15–16):527–32. 10.1007/s00508-017-1179-x 28236043

[2] Brown SD White R Tobin P. Keep them breathing: cystic fibrosis pathophysiology, diagnosis, and treatment. JAAPA. (2017) 30(5):23–7. 10.1097/01.JAA.0000515540.36581.92 28441669

[3] Grasemann H Ratjen F. Early lung disease in cystic fibrosis. Lancet Respir Med. (2013) 1(2):148–57. 10.1016/S2213-2600(13)70026-2 24429095

[4] Ramsey KA Ranganathan S Park J Skoric B Adams AM Simpson SJ Early respiratory infection is associated with reduced spirometry in children with cystic fibrosis. Am J Respir Crit Care Med. (2014) 190(10):1111–6. 10.1164/rccm.201407-1277OC 25321321

[5] Breuer O Caudri D Stick S Turkovic L. Predicting disease progression in cystic fibrosis. Expert Rev Respir Med. (2018) 12(11):905–17. 10.1080/17476348.2018.1519400 30173593

[6] Castellani C Duff AJA Bell SC Heijerman HGM Munck A Ratjen F ECFS best practice guidelines: the 2018 revision. J Cyst Fibros. (2018) 17(2):153–78. 10.1016/j.jcf.2018.02.006 29506920

[7] Burgel PR Southern KW Addy C Battezzati A Berry C Bouchara JP Standards for the care of people with cystic fibrosis (CF); recognising and addressing CF health issues. J Cyst Fibros. (2024) 23(2):187–202. 10.1016/j.jcf.2024.01.005 38233247

[8] Ranganathan S Linnane B Nolan G Gangell C Hall G. Early detection of lung disease in children with cystic fibrosis using lung function. Paediatr Respir Rev. (2008) 9(3):160–7. 10.1016/j.prrv.2008.05.002 18694707

[9] Lahiri T Hempstead SE Brady C Cannon CL Clark K Condren ME Clinical practice guidelines from the cystic fibrosis foundation for preschoolers with cystic fibrosis. Pediatrics. (2016) 137(4):e20151784. 10.1542/peds.2015-1784 27009033

[10] Soni R Dobbin CJ Milross MA Young IH Bye PPT. Gas exchange in stable patients with moderate-to-severe lung disease from cystic fibrosis. J Cyst Fibros. (2008) 7(4):285–91. 10.1016/j.jcf.2007.11.003 18785322

[11] Zavorsky GS Cao J Mayo NE Gabbay R Murias JM. Arterial versus capillary blood gases: a meta-analysis. Respir Physiol Neurobiol. (2007) 155(3):268–79. 10.1016/j.resp.2006.07.002 16919507

[12] Pitkin AD Roberts CM Wedzicha JA. Arterialised earlobe blood gas analysis: an underused technique. Thorax. (1994) 49(4):364–6. 10.1136/thx.49.4.364 8202909 PMC475372

[13] Gaultier C Boule M Allaire Y Clement A Buvry A Girard F. Determination of capillary oxygen tension in infants and children: assessment of methodology and normal values during growth. Bull Eur Physiopathol Respir. (1979) 14(3):287–97. 476326

[14] Kraemer R Latzin P Pramana I Ballinari P Gallati S Frey U. Long-term gas exchange characteristics as markers of deterioration in patients with cystic fibrosis. Respir Res. (2009) 10(1):106. 10.1186/1465-9921-10-106 19909502 PMC2780404

[15] Fuger M Aupiais C Thouvenin G Taytard J Tamalet A Escudier E Gas exchanges in children with cystic fibrosis or primary ciliary dyskinesia: a retrospective study. Respir Physiol Neurobiol. (2018) 251:1–7. 10.1016/j.resp.2018.01.010 29366817

[16] Graham BL Steenbruggen I Miller MR Barjaktarevic IZ Cooper BG Hall GL Standardization of spirometry 2019 update. An official American thoracic society and European respiratory society technical statement. Am J Respir Crit Care Med. (2019) 200(8):e70–88. 10.1164/rccm.201908-1590ST 31613151 PMC6794117

[17] Quanjer PH Stanojevic S Cole TJ Baur X Hall GL Culver BH Multi-ethnic reference values for spirometry for the 3–95-yr age range: the global lung function 2012 equations. Eur Respir J. (2012) 40(6):1324–43. 10.1183/09031936.00080312 22743675 PMC3786581

[18] Robinson PD Latzin P Verbanck S Hall GL Horsley A Gappa M Consensus statement for inert gas washout measurement using multiple- and single- breath tests. Eur Respir J. (2013) 41(3):507–22. 10.1183/09031936.00069712 23397305

[19] Anagnostopoulou P Latzin P Jensen R Stahl M Harper A Yammine S Normative data for multiple breath washout outcomes in school-aged Caucasian children. Eur Respir J. (2020) 55(4):1901302. 10.1183/13993003.01302-2019 31862765

[20] Kentgens AC Latzin P Anagnostopoulou P Jensen R Stahl M Harper A Normative multiple-breath washout data in school-aged children corrected for sensor error. Eur Respir J. (2022) 60(2):2102398. 10.1183/13993003.02398-2021 35710262

[21] Proesmans M Balinska-Miskiewicz W Dupont L Bossuyt X Verhaegen J Hoiby N Evaluating the “Leeds criteria” for pseudomonas aeruginosa infection in a cystic fibrosis centre. Eur Respir J. (2006) 27(5):937–43. 10.1183/09031936.06.00100805 16707392

[22] Stevens DA Moss RB Kurup VP Knutsen AP Greenberger P Judson MA Allergic bronchopulmonary aspergillosis in cystic fibrosis–state of the art. Cystic fibrosis foundation consensus conference. Clin Infect Dis. (2003) 37(Suppl 3):S225–64. 10.1086/376525 12975753

[23] McKone EF Goss CH Aitken ML. CFTR genotype as a predictor of prognosis in cystic fibrosis. Chest. (2006) 130(5):1441–7. 10.1378/chest.130.5.1441 17099022

[24] Krivchenia K Hawkins SM Iyer NP Hayes D Jr Deterding RR Ruminjo J 2019 clinical practice guideline summary for clinicians: home oxygen therapy for children. Ann Am Thorac Soc. (2019) 16(7):781–5. 10.1513/AnnalsATS.201902-136CME 30990761

[25] Tittle M Flynn MB. Correlation of pulse oximetry and co-oximetry. Dimens Crit Care Nurs. (1997) 16(2):88–95. 10.1097/00003465-199703000-00004 9104146

[26] Collins JA Rudenski A Gibson J Howard L O’Driscoll R. Relating oxygen partial pressure, saturation and content: the haemoglobin-oxygen dissociation curve. Breathe (Sheff). (2015) 11(3):194–201. 10.1183/20734735.001415 26632351 PMC4666443

[27] Nitzan M Romem A Koppel R. Pulse oximetry: fundamentals and technology update. Med Devices (Auckl). (2014) 7:231–9. 25031547 10.2147/MDER.S47319 PMC4099100

[28] VanDevanter DR Wagener JS Pasta DJ Elkin E Jacobs JR Morgan WJ Pulmonary outcome prediction (POP) tools for cystic fibrosis patients. Pediatr Pulmonol. (2010) 45(12):1156–66. 10.1002/ppul.21311 20717915 PMC4112577

[29] Orenti A Zolin A Jung A van Rens J Prasad V Fox A ECFSPR annual report 2021. (2023). Available online at: https://www.ecfs.eu/sites/default/files/Annual%20Report_2021_09Jun2023.pdf (Accessed August 26, 2023).

[30] Vilozni D Bentur L Efrati O Minuskin T Barak A Szeinberg A Spirometry in early childhood in cystic fibrosis patients. Chest. (2007) 131(2):356–61. 10.1378/chest.06-1351 17296633

[31] Kraemer R Blum A Schibler A Ammann RA Gallati S. Ventilation inhomogeneities in relation to standard lung function in patients with cystic fibrosis. Am J Respir Crit Care Med. (2005) 171(4):371–8. 10.1164/rccm.200407-948OC 15531750

[32] Urquhart DS Montgomery H Jaffe A. Assessment of hypoxia in children with cystic fibrosis. Arch Dis Child. (2005) 90(11):1138–43. 10.1136/adc.2005.071795 16243867 PMC1720198

[33] Aurora P Bush A Gustafsson P Oliver C Wallis C Price J Multiple-breath washout as a marker of lung disease in preschool children with cystic fibrosis. Am J Respir Crit Care Med. (2005) 171(3):249–56. 10.1164/rccm.200407-895OC 15516530

[34] Aurora P Stanojevic S Wade A Oliver C Kozlowska W Lum S Lung clearance index at 4 years predicts subsequent lung function in children with cystic fibrosis. Am J Respir Crit Care Med. (2011) 183(6):752–8. 10.1164/rccm.200911-1646OC 20935113

[35] Davies G Aurora P. The use of multiple breath washout for assessing cystic fibrosis in infants. Expert Rev Respir Med. (2017) 11(1):21–8. 10.1080/17476348.2017.1269604 27927050

