# Supplementary material for: Arterialized oxygen tension and unfavorable clinical outcomes in pediatric cystic fibrosis
Source: Front Pediatr. 2025 Oct 8;13:1653323. doi: 10.3389/fped.2025.1653323 (PMC12540158; doi:10.3389/fped.2025.1653323)
Supplement: Supplementary file 1 [file Datasheet1.docx]

**Supplementary materials**

**Supplementary Tables**

**TABLE S1. Patient characteristics and examinations categorized by age group**

| Age  [years] | **5** | **6** | **7** | **8** | **9** | **10** | **11** | **12** | **13** | **14** | **15** | **16** | **17** | **18** |
| --- | --- | --- | --- | --- | --- | --- | --- | --- | --- | --- | --- | --- | --- | --- |
| **Spirometry [N]** | 96 | 99 | 94 | 91 | 86 | 81 | 73 | 64 | 55 | 51 | 42 | 37 | 30 | 24 |
| FEV1^a^  [Z-scores] | -0.09 (1.06) | 0.05 (1.13) | -0.01 (1.05) | -0.11 (1.00) | -0.13 (1.11) | -0.33 (1.04) | -0.56 (1.06) | -0.71 (1.19) | -0.86 (1.27) | -0.70 (1.32) | -0.69 (1.43) | -0.81 (1.63) | -1.31 (1.75) | -1.55 (1.70) |
| **BGA**  **[N]** | 96 | 99 | 94 | 91 | 86 | 81 | 73 | 64 | 55 | 51 | 42 | 37 | 30 | 24 |
| pO_2_^a^  [mmHg] | 87.2 (8.0) | 87.3 (8.1) | 87.2 (8.4) | 87.3 (7.3) | 86.2 (7.1) | 86.4 (7.0) | 87.9 (7.4) | 86.7 (7.7) | 85.5 (7.7) | 86.4 (7.2) | 87.2 (8.2) | 87.6 (7.7) | 86.9 (7.3) | 88.3 (8.0) |
| pO_2_^a^  [kPa] | 11.6 (1.1) | 11.6 (1.1) | 11.6 (1.1) | 11.6 (1.0) | 11.5 (0.9) | 11.5 (0.9) | 11.7 (1.0) | 11.6 (1.0) | 11.4 (1.0) | 11.5 (1.0) | 11.6 (1.1) | 11.7 (1.0) | 11.6 (1.0) | 11.8 (1.1) |
| pO_2_^a^  [Z-score] | -0.33  (1.45) | -0.30  (1.47) | -0.32  (1.52) | -1.07  (1.41) | -1.28  (1.39) | -1.23  (1.36) | -0.95  (1.44) | -1.19  (1.50) | -1.41  (1.50) | -1.24  (1.40) | -1.08  (1.59) | -1.01  (1.41) | -1.15  (1.41) | -0.88  (1.54) |
| **N_2_MBW [N]** | NA | 9 | 15 | 21 | 21 | 21 | 21 | 11 | 14 | 11 | 9 | 9 | 15 | 11 |
| LCI_2.5_^a^ | NA | 7.4 (1.4) | 9.7 (2.5) | 10.1 (2.9) | 9.6 (3.6) | 9.0 (2.4) | 10.0 (3.2) | 10.9 (3.8) | 10.1 (3.2) | 9.9 (3.1) | 9.7 (3.8) | 10.3 (4.0) | 12.5 (5.7) | 12.5 (5.6) |
| **Modulator** | 0/96  (0%) | 1/99  (1%) | 1/94  (1%) | 1/91  (1%) | 1/86  (1%) | 6/81  (7%) | 9/73  (12%) | 7/64  (11%) | 9/55  (16%) | 8/51  (16%) | 7/42  (17%) | 5/37  (14%) | 2/30  (7%) | 6/24^b^  (25%) |
| Dual [n] | 0 | 1 | 1 | 1 | 0 | 5 | 8 | 6 | 7 | 6 | 5 | 3 | 1 | 5 |
| HEMT [n] | 0 | 0 | 0 | 0 | 1 | 1 | 1 | 1 | 2 | 2 | 2 | 2 | 1 | 1 |
| **Modulator use dichotomized by pO₂ at age 5 years [Z-score]** | | | | | | | |  |  |  |  |  |  |  |
| < -1.64 [n] | NA | 0 | 0 | 0 | 0 | 0 | 1 | 2 | 3 | 2 | 3 | 3 | 0 | 1 |
| ≥ -1.64 [n] | NA | 1 | 1 | 1 | 1 | 6 | 8 | 5 | 6 | 6 | 4 | 2 | 2 | 4 |
| **chronic PsA** | 4/96  (4%) | 2/99  (2%) | 4/94  (4%) | 5/91  (6%) | 6/86  (7%) | 5/81  (6%) | 7/73  (10%) | 11/64  (17%) | 8/55  (15%) | 12/51  (24%) | 10/42  (24%) | 6/37  (16%) | 6/30  (20%) | 8/24  (33%) |
| If not otherwise indicated the number of patients & proportion [n/N (%)] are reported  ^a^ Mean (SD)  ^b^ For one individual with CF, data at 5 years of age was not available  The examinations for the same individual within the same age group were conducted on the same date.  Abbreviations used: N (number of patients), FEV1 (forced expiratory volume in the first second), BGA (blood gas analysis), kPa (kilo pascal), mmHg (millimeters of mercury), N_2_MBW (nitrogen multiple breath washout), LCI_2.5_ (lung clearance index), NA (not applicable), pO_2_ (arterialized oxygen tension), PsA (Pseudomonas aeruginosa), Dual (ivacaftor/lumacaftor or ivacaftor/tezacaftor), HEMT (Highly Effective Modulator Therapy). | | | | | | | | | | | | | | |

**TABLE S2. Patients’ characteristics at the age of 5 years**

|  |  | ***pO_2_ at 5 years [Z-score]*** | | ***p-value*** |
| --- | --- | --- | --- | --- |
|  |  | ***< -1.64***^a^ | ***≥ -1.64***^a^ |  |
| ***Number of patients (N)*** | ***96*** | ***19*** | ***77*** |  |
| Sex |  |  |  | 0.091 |
| female |  | 12 (63%) | 32 (42%) |  |
| male |  | 7 (37%) | 45 (58%) |  |
| Genotype |  |  |  | n.s |
| high-risk |  | 17 (89%) | 66 (86%) |  |
| low-risk |  | 2 (11%) | 6 (7.5%) |  |
| unknown |  | 0 (0%) | 5 (6.5%) |  |
| Pancreatic insufficiency |  | 17 (89%) | 66 (86%) | n.s. |
| FEV1 [Z-score]^b^  FEV1 [% predicted]^b^ |  | -0.67 (1.21)  91.2 (15.5) | 0.05 (0.97)  100.6 (12.4) | 0.021 |
| ***Clinical measures < 5 years (N)*** |  | ***19*** | ***76***^c^ |  |
| chronic PsA infection |  | 3 (15.8%) | 7 (9.2%) | n.s. |
| ABPA |  | 0 0%) | 1 (1.3%) | n.s. |
| CFRD |  | 0 (0%) | 0 (0%) | n.s. |

If not otherwise indicated count & proportion [n (%)] were reported

^a^ A pO₂ Z-score of -1.64 corresponds to 80 mmHg at the age of 5 years

^b^ Mean (SD)

^c^ One patient was transferred to our center at the age of 5

Risk stratification within genotypes was determined based on CFTR mutations and functional phenotypes. CFTR mutations were categorized as high-risk if both alleles featured class I, class II, or class III mutations, while class IV and V mutations were classified as low-risk; otherwise, they were designated as unknown (E1).

Abbreviations used: N (number of patients), FEV1 (forced expiratory volume in the first second), PsA (Pseudomonas aeruginosa), ABPA (allergic bronchopulmonary aspergillosis), CFRD (CF-related Diabetes), pO_2_ (arterialized oxygen tension).

**TABLE S3. Number of patients analyzed for CF-related complications**

|  |  | ***pO_2_ at 5 years [Z-score]*** | |
| --- | --- | --- | --- |
|  |  | ***< -1.64^b^*** | ***≥ -1.64^b^*** |
| ***Number of patients in total (N)*** | ***95^a^*** | ***19*** | ***76^a^*** |
| ***Excluded from analysis*** |  |  |  |
| chronic PsA infection |  | 3/19 (15.8%) | 7/76 (9.2%) |
| ABPA |  | 0/19 (0.0%) | 1/76 (1.3%) |
| CFRD |  | 0/19 (0.0%) | 0/76 (0.0%) |
| ***Observed complications from 5 to 18 years*** | | |  |
| chronic PsA infection |  | 7/16 (43.8%) | 9/69 (13.0%) |
| ABPA |  | 7/19 (36.8%) | 4/75 (5.3%) |
| CFRD |  | 7/19 (37.0%) | 7/76 (9.1%) |

If not otherwise indicated count & proportion [n (%)] were reported

^a^ One patient was transferred to our center at the age of 5

^b^ A pO₂ Z-score of -1.64 corresponds to 80 mmHg at the age of 5 years

Presented are the numbers of patients excluded from analysis due to early acquisition (before the age of 5 years) and the observed cystic fibrosis-related complications between the ages of 5 and 18 years.

Abbreviations used: N (number of patients), PsA (Pseudomonas aeruginosa), ABPA (allergic bronchopulmonary aspergillosis), CFRD (CF-related Diabetes), pO_2_ (arterialized oxygen tension).

**Supplementary statistics**

**TABLE S4. Simple linear mixed models investigating the association of potential factors on the time course of FEV1**

| **Effect** | **Comparison**  [? vs. Ref.] | **Estimate**  [Z-score] | **lower CI** | **upper CI** | **p-value** |
| --- | --- | --- | --- | --- | --- |
| **Simple models without interaction terms** | | | | | |
| Group | <-1.64 vs. ≥-1.64 | -0.62 | -1.12 | -0.12 | 0.015 |
| Age [years] |  | -0.13 | -0.15 | -0.12 | <0.001 |
| Sex | male vs. female | -0.05 | -0.47 | 0.37 | 0.820 |
| Pancreatic insufficiency (PI) | yes vs. no | -0.72 | -1.31 | -0.12 | 0.018 |
| Genotype risk | low vs. high | -1.01 | -1.75 | -0.27 | 0.008 |
| FEV1 at age 5 [Z-score] |  | 0.73 | 0.59 | 0.86 | <0.001 |
| Year of birth - calendar year |  | 0.05 | 0.01 | 0.10 | 0.021 |
| BMI at age 5 | normal vs. low ^1^ | -0.50 | -0.94 | -0.07 | 0.023 |
| Chronic PsA < 5 | no vs. yes | -0.35 | -1.05 | 0.34 | 0.320 |
| Chronic Staph. aures < 5 | no vs. yes | 0.17 | -0.52 | 0.87 | 0.627 |
| 1^st^ PsA infection < 5 | no vs. yes | -0.78 | -1.19 | -0.36 | <0.001 |
| CFTR modulator use | yes vs. no | -0.44 | -0.69 | -0.19 | 0.001 |
|  |  |  |  |  |  |
| **Simple models including interaction terms** | | | | | |
| Interaction Group * Age | <-1.64 vs. ≥-1.64 | -0.06 | -0.09 | -0.02 | <0.001 |
| Interaction Sex * Age | male vs. female | 0.03 | -0.00 | 0.06 | 0.059 |
| Interaction PI * Age | yes vs. no | -0.10 | -0.15 | -0.05 | 0.013 |
| Interaction Genotype risk * Age | low vs. high | -0.14 | -0.15 | -0.12 | <0.001 |
| BMI at age 5 * Age | normal vs. low ^1^ | -0.01 | -0.04 | 0.03 | 0.752 |
| Interaction chronic PsA < 5 * Age | no vs. yes | -0.09 | -0.13 | -0.04 | <0.001 |
| Interaction chronic Staph. aureus < 5 * Age | no vs. yes | 0.00 | -0.05 | 0.04 | 0.870 |
| Interaction 1^st^ PsA infection < 5 * Age | no vs. yes | -0.04 | -0.08 | -0.01 | 0.019 |
|  | | | | | |
| **Multivariable model on FEV1 (Z-score) time course** | | | | | |
| Interaction Group * Age | <-1.64 vs. ≥-1.64 | -0.04 | -0.07 | -0.01 | 0.015 |
| Interaction PI * Age | yes vs. no | -0.17 | -0.46 | 0.13 | 0.264 |
| Interaction Genotype risk * Age | low vs. high | 0.10 | -0.20 | 0.40 | 0.496 |
| FEV1 at age 5 [Z-score] |  | 0.78 | 0.64 | 0.93 | <0.001 |
| Year of birth - calendar year |  | 0.02 | -0.02 | 0.05 | 0.281 |
| CFTR modulator use | yes vs. no | 0.19 | -0.03 | 0.40 | 0.088 |
| Interaction chronic PsA < 5 * Age | no vs. yes | -0.07 | -0.12 | -0.03 | 0.002 |

^1^ BMI-adjusted equivalent was used as a binary variable (≥20 kg/m² vs. <20 kg/m²), since only one individual had a Z-score below -2

The Group variable was defined based on pO_2_ values at the age of 5 years, and all metric outcomes collected thereafter were grouped according to the baseline grouping.

The confounders were selected based on their known influence on the FEV1 course in individuals with Cystic Fibrosis. The variable ‘Year of birth’ was included to account for the improving prognosis of individuals with Cystic Fibrosis over the past decades, reflecting advancements in therapy and quality of care.

In accordance with the simple models, a multivariable model was constructed. This multivariable model incorporated the following factors: Group, age, pancreatic insufficiency, genotype risk, year of birth, FEV1 at age 5, chronic Pseudomonas aeruginosa infection before age 5, and CFTR modulator use along with their respective interaction terms that demonstrated statistical significance in the simple models.

The results indicate that individuals with pO_2_ Z-scores below -1.64 (= 80 mmHg) at age 5 experienced a significantly larger decrease in their lung function with increasing age, as compared to normoxemic patients at baseline (Estimate Interaction term [Z-score*year^-1^]: -0.04, CI: -0.07 – -0.01, p = 0.015).

**Supplementary Figures**

**FIGURE S1. Flow chart**

* Insufficient data: patients were either transferred to our CF center or diagnosed after the age of 5, with inconsistent records from earlier periods.

** No available surveillance data for the period from birth to age 5.

PwCF born and transplanted during the inclusion period (October 1997 – December 2020): N = 26, with none receiving care at our CF center for at least one year prior to transplantation.

Multiple breath washout (MBW) maneuvers have been routinely performed at our CF center since 2015. Due to the limited number of measurements, a cross-sectional design was chosen for the MBW analysis, incorporating all measurements conducted since the method's implementation (N of measurements = 188).

Abbreviations used: ABPA (allergic bronchopulmonary aspergillosis), CFRD (CF-related diabetes), cPsA (chronic Pseudomonas aeruginosa infection), N (number of patients), pO_2_ (arterialized oxygen tension), PwCF (people with CF).


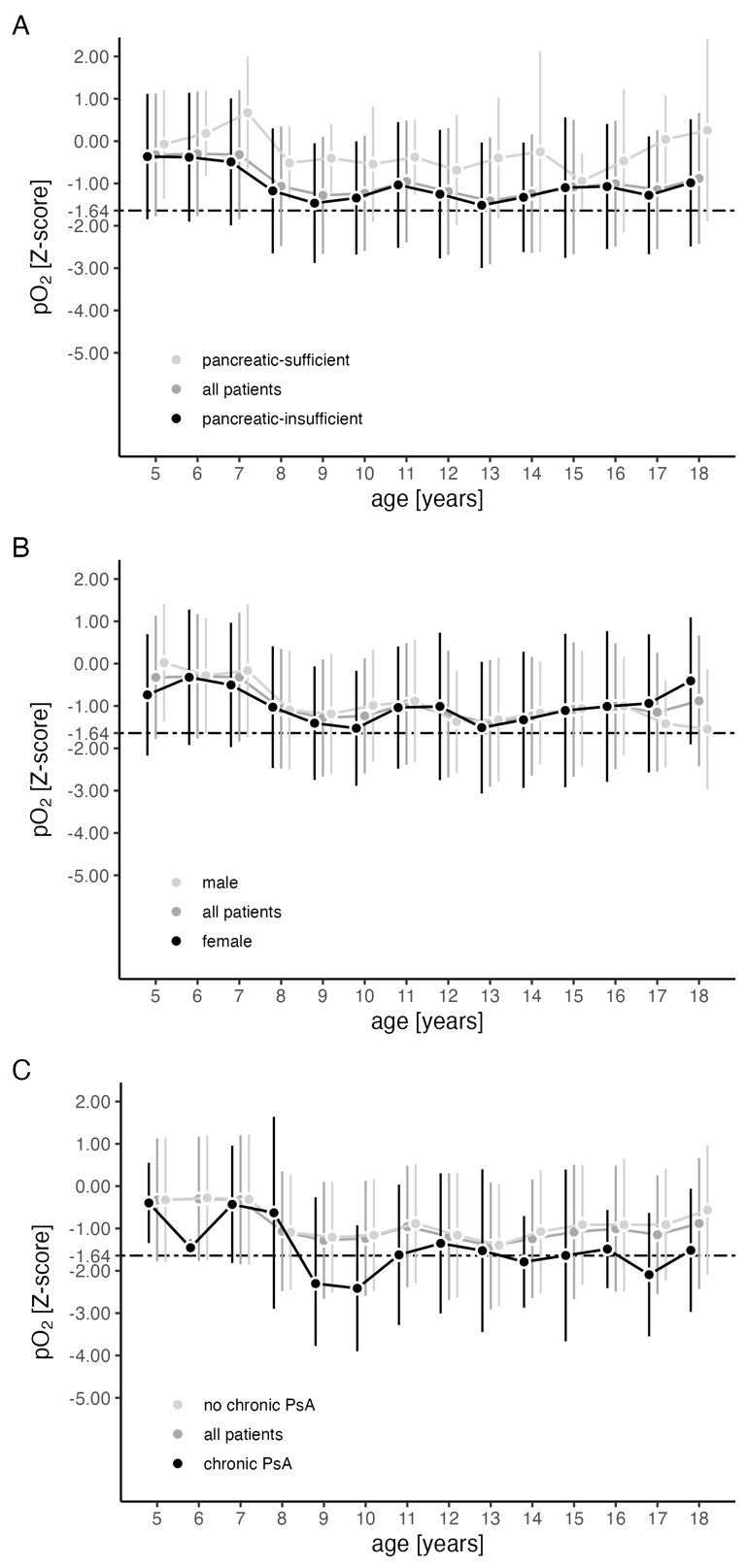


**FIGURE S2. Associations between pO_2_ trajectories and clinical factors**

Mean values (points) and standard deviations (vertical lines) of arterialized oxygen tension (pO₂) trajectories are shown across age groups for all patients (grey) and stratified by pancreatic function (A), sex (B), and chronic Pseudomonas aeruginosa (PsA) infection (C). The black dash-dot line indicates the lower limit of normal (LLN) for pO₂, corresponding to a Z-score of -1.64 (5-8 years: 80 mmHg; ≥8 years: 84.3 mmHg).


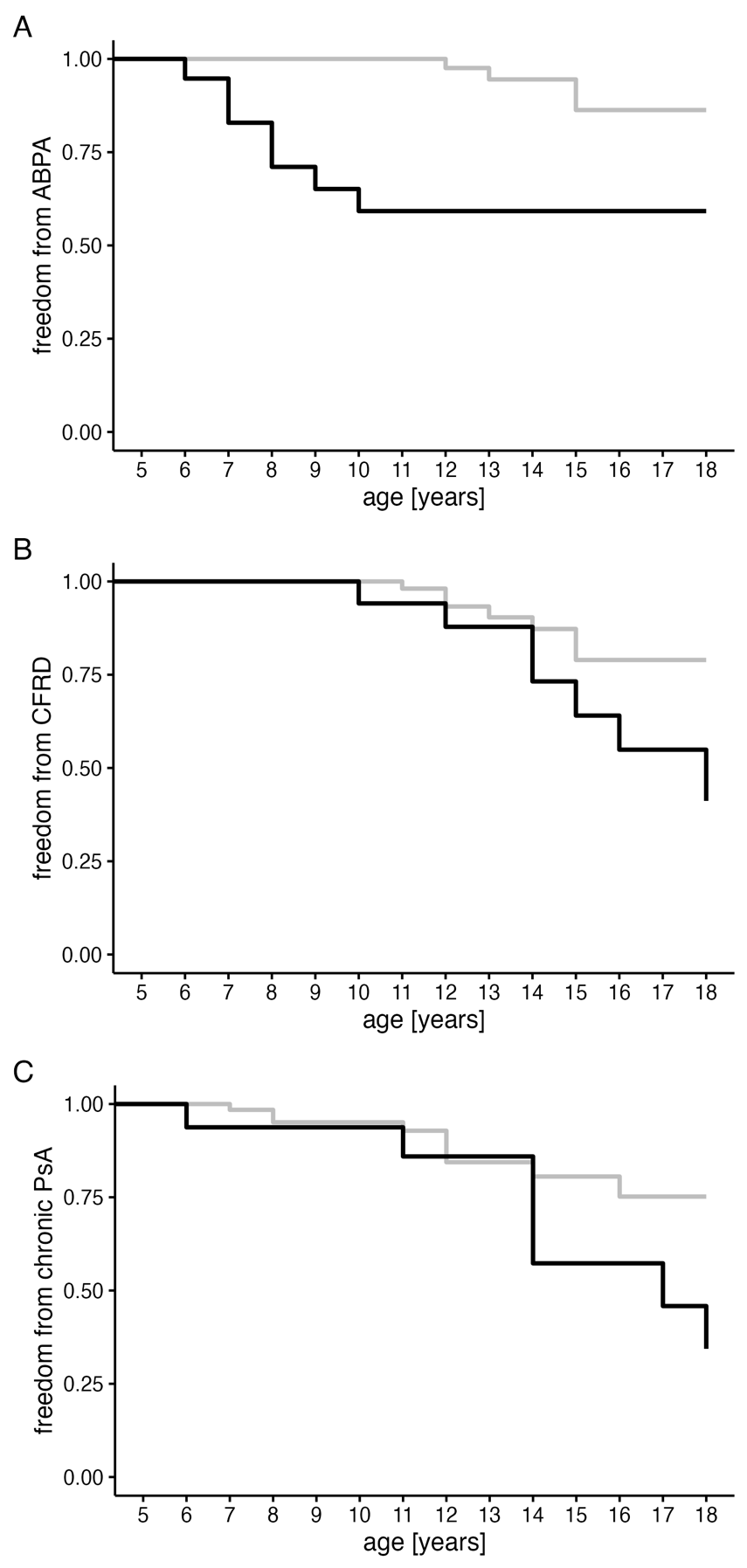


**FIGURE S3. Onset of CF-related complications in relation to pO_2_ at age 5**

The probability of freedom from (A) allergic bronchopulmonary aspergillosis (ABPA), (B) CF-related diabetes (CFRD) and (C) chronic Pseudomonas aeruginosa (PsA) infection until transition to adult ward is illustrated separately for individuals with early abnormal pO_2_ (Z-score < -1.64, black line) and those with normal pO_2_ (Z-score ≥ -1.64, grey line).

**Supplementary References**

S1. McKone EF, Goss CH, Aitken ML. CFTR genotype as a predictor of prognosis in cystic fibrosis. Chest. 2006;130(5):1441-7.
